# Supplementary material for: DMRforPairs: identifying Differentially Methylated Regions between unique samples using array based methylation profiles
Source: BMC Bioinformatics. 2014 May 15;15:141. doi: 10.1186/1471-2105-15-141 (PMC4046028; doi:10.1186/1471-2105-15-141)
Supplement: Additional file 2 — DMRforPairs output for the comparison of A431-MCF7 and NA17018-NA17105. Please start from the HTML files in each folder. Available via the BMC Bioinformatics website. [file 1471-2105-15-141-S2.zip › 1394847754114233_MOESM2_ESM/A431_MCF7/figures/17955.pdf]

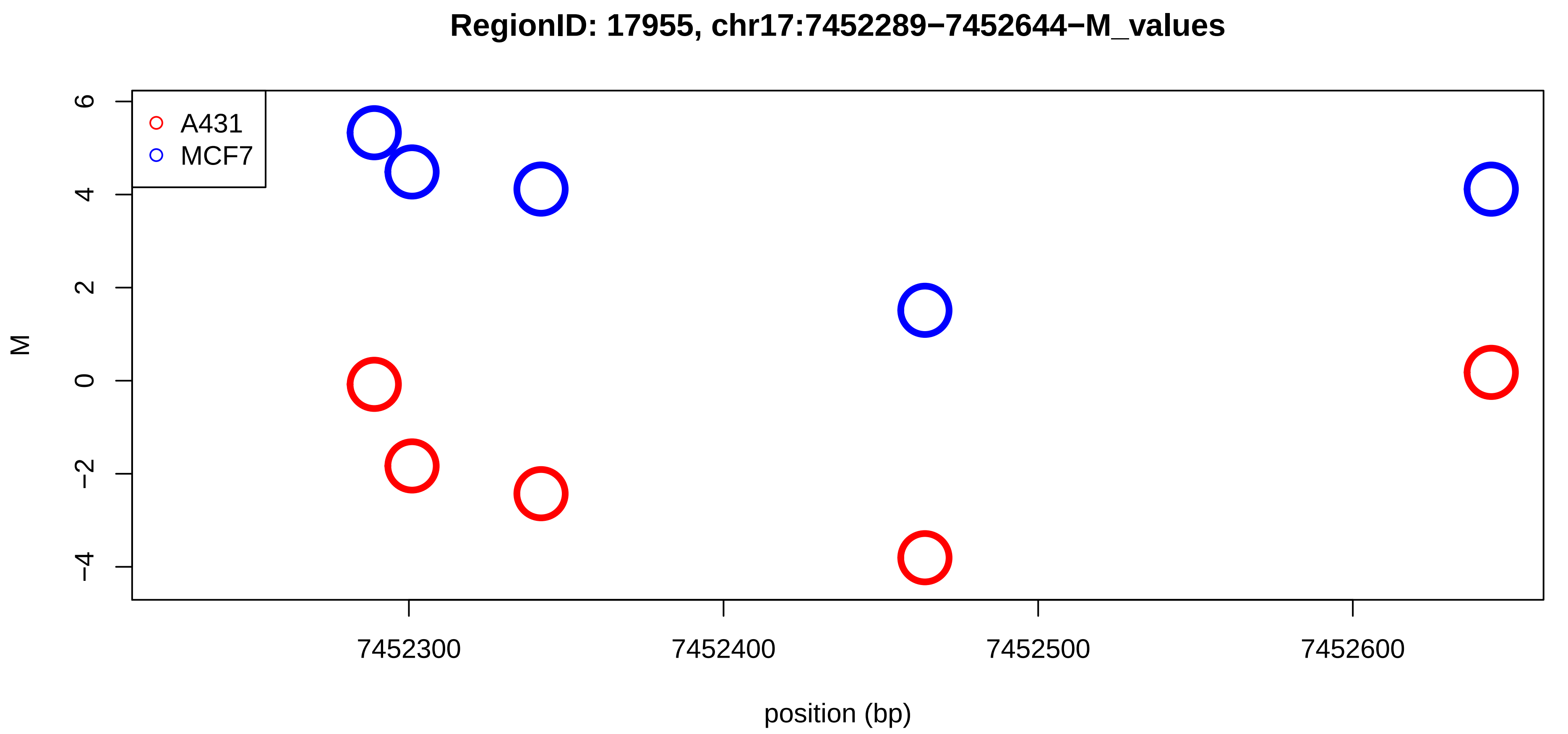

RegionID: 17955, chr17:7452289−7452644−M_values
A431
MCF7
M
6
4
2
0
−2
−4
7452300
7452400
7452500
7452600
position (bp)

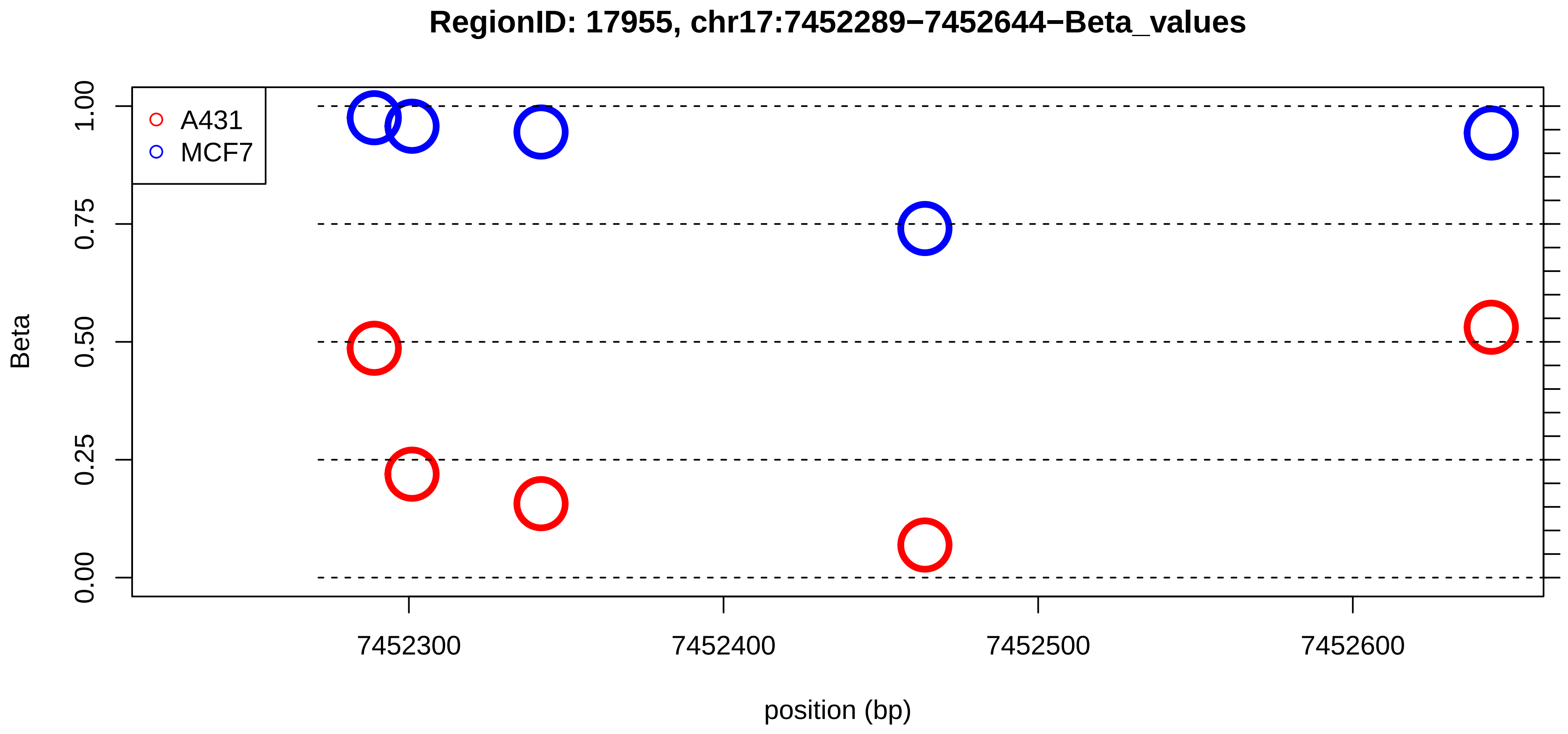

RegionID: 17955, chr17:7452289−7452644−Beta_values
A431
MCF7
Beta
1.00
0.75
0.50
0.25
0.00
7452300
7452400
7452500
7452600
position (bp)

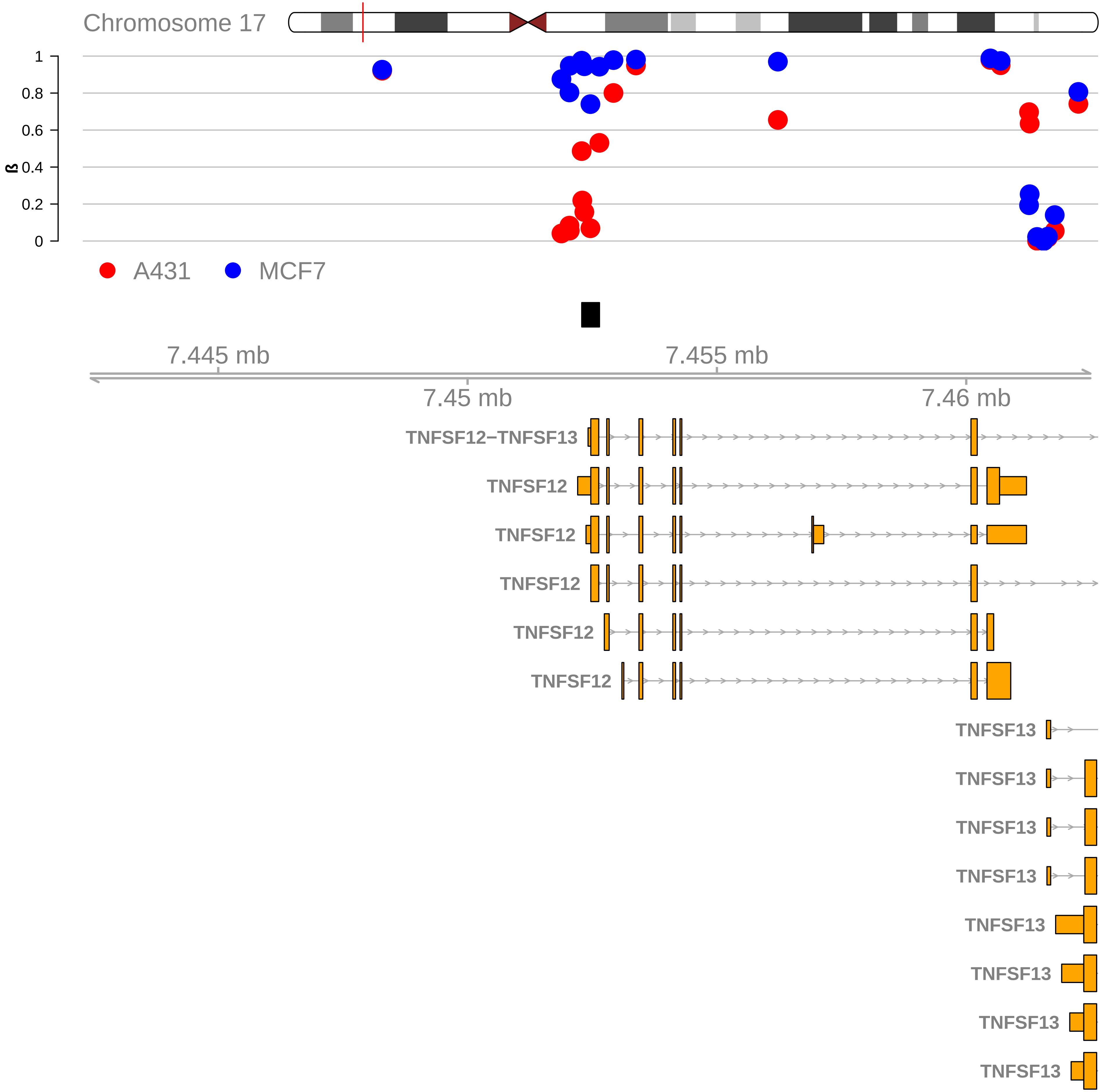

Chromosome 17
1
0.8
0.6
ß 0.4
0.2
0
A431
MCF7
7.445 mb
7.455 mb
7.45 mb
7.46 mb
TNFSF12−TNFSF13
TNFSF12
TNFSF12
TNFSF12
TNFSF12
TNFSF12
TNFSF13
TNFSF13
TNFSF13
TNFSF13
TNFSF13
TNFSF13
TNFSF13
TNFSF13
